# Supplementary material for: A variational expectation-maximization framework for balanced multi-scale learning of protein and drug interactions
Source: Nat Commun. 2024 May 25;15:4476. doi: 10.1038/s41467-024-48801-4 (PMC11530528; doi:10.1038/s41467-024-48801-4)
Supplement: Supplementary file 1 — Supplementary Information [file 41467_2024_48801_MOESM1_ESM.pdf]

Supplementary information for A Variational Expectation-Maximization Framework for Balanced Multi-scale Learning of Protein and Drug Interactions

Supplementary Methods

Balanced Optimization Algorithm

The workflow of the EM algorithm is summarized in Algorithm 1. The optimization process iteratively performs the E-step and the M-step. During the M-step, the atomic structure scale model GNNϕ generates both atomic structural embeddings and pseudo-labels, which are utilized for training the molecular network scale model GNN_θ. These embeddings are treated as node initial attributes, while the pseudo-labels serve as pseudo edges for link prediction. In the E-step, the molecular network scale model GNN_θ is trained using both the pseudo-labels predicted by the atomic structure scale model GNN_ϕ and the observed labels.

Algorithm 1 Expectation-maximization algorithm

```
1: Input: A multi-scale network N, a few labeled interactions y_{L_V}.
2: Output: Unknown interactions labels y_{L_U} for the missing interactions L_V.
3: while not coverage do
4:     E-step: Atomic Structure scale Modeling
5:     Annotate unlabeled interactions with p_θ.
6:     Set y_L = (y_{L_V}, y_{L_U}) and update q_φ with Eq. 11 and GNN_φ.
7:     M-step: Molecular network scale Modeling.
8:     Annotate unlabeled interactions with q_φ and y_L.
9:     Update p_θ with Eq. 14 based on GNN_θ.
10: end while
```

Optimality Condition for q_ϕ

Recall that our goal of Eq. 5 for q_ϕ is to minimize the KL divergence $q_\phi(\mathbf{y}_{\mathcal{L}_U}|\mathcal{G}, \mathcal{D})$ (e.g. simplifying as $q_\phi(\mathbf{y}_{\mathcal{L}_U})$) between $p_\theta(\mathbf{y}_{\mathcal{L}_U}|\mathbf{y}_{\mathcal{L}_V}, \mathcal{G}, A)$ (e.g. simplifying as $p_\theta(\mathbf{y}_{\mathcal{L}_U}|\mathbf{y}_{\mathcal{L}_V})$). Therefore, following the idea of GMNN [1], the objective function for q_ϕ could be formulated as follows:

$$\begin{aligned}\mathcal{O}(q_\phi(\mathbf{y}_{\mathcal{L}_U})) &= -\mathrm{KL}\left(q_\phi(\mathbf{y}_{\mathcal{L}_U})||p_\theta(\mathbf{y}_{\mathcal{L}_U}|\mathbf{y}_{\mathcal{L}_V})\right)\\ &= \sum_{\mathbf{y}_{\mathcal{L}_U}} q_\phi(\mathbf{y}_{\mathcal{L}_U})\left[\log p_\theta(\mathbf{y}_{\mathcal{L}_U}|\mathbf{y}_{\mathcal{L}_V}) - \log q_\phi(\mathbf{y}_{\mathcal{L}_U})\right]\\ &= \sum_{\mathbf{y}_{\mathcal{L}_U}}\left(\prod_{L_{ij}} q_\phi(\mathbf{y}_{L_{ij}})\right)\left[\log p_\theta(\mathbf{y}_{\mathcal{L}_U}|\mathbf{y}_{\mathcal{L}_V}) - \sum_{L_{ij}}\log q_\phi(\mathbf{y}_{\mathcal{L}_{L_{ij}}})\right] + \mathrm{const}\\ &= \sum_{\mathbf{y}_{L_{ij}}}\sum_{\mathbf{y}_{\mathcal{L}_U\setminus L_{ij}}}\left(q_\phi(\mathbf{y}_{L_{ij}})\prod_{L'\neq L_{ij}} q_\phi(\mathbf{y}_{L'})\right)\left[\log p_\theta(\mathbf{y}_{\mathcal{L}_U}|\mathbf{y}_{\mathcal{L}_V}) - \sum_{L'}\log q_\phi(\mathbf{y}_{\mathcal{L}'})\right] + \mathrm{const}\\ &= \sum_{\mathbf{y}_{L'}}\log\mathcal{F}(\mathbf{y}_{\mathcal{L}'}) - \sum_{\mathbf{y}_{L'}}\log q_\phi(\mathbf{y}_{\mathcal{L}'}) + \mathrm{const}\\ &= -\mathrm{KL}\left(q_\phi(\mathbf{y}_{\mathcal{L}'})||\frac{\mathcal{F}(\mathbf{y}_{\mathcal{L}'})}{Z}\right) + \mathrm{const}\end{aligned} \tag{1}$$

where Z is a normalization term and $\mathcal{F}(\mathbf{y}_{\mathcal{L}'})$ is the valid distribution on $\mathbf{y}_{\mathcal{L}'}$:

$$\begin{aligned}\mathcal{F}(\mathbf{y}_{\mathcal{L}'}) &= \sum_{\mathbf{y}_{\mathcal{L}_U\setminus L_{ij}}}\prod_{L'\neq L_{ij}} q_\phi(\mathbf{y}_{\mathcal{L}'})\log p_\theta(\mathbf{y}_{\mathcal{L}_U},\mathbf{y}_{\mathcal{L}_V})\\ &= \mathbb{E}_{q_\phi(\mathbf{y}_{\mathcal{L}_U\setminus L_{ij}})}\left[\log\left(\mathbf{y}_{\mathcal{L}_U},\mathbf{y}_{\mathcal{L}_V}\right)\right]\end{aligned} \tag{2}$$

Supplementary Notes

1 Implementation details

Here we describe the implementation details for the atomic structure scale and molecular network scale modeling.

1.1 Protein and Drug Interaction Prediction

1.1.1 Protein-Protein Interactions

Datasets and Baselines

For the protein-protein interactions (PPIs), we employed the data splitting approach proposed by previous studies [2, 3], and the data statistics are presented in Table 1. Specifically, we evaluated our model along with baseline methods using the random split (Random) and the partition schemes based on Breadth-First Search (BFS) and Depth-First Search (DFS).

We included two single-scale methods (DrugVQA [4], TAG-PPI [5]) as well as two multi-scale GNN models (GNN-PPI [3], HIGH-PPI [2]) as our baselines. The **DrugVQA** model operates at the atomic structure scale, predicting interactions between proteins using their contact maps as features. **TAG-PPI** predicts PPIs by employing a 1D convolution on protein sequences and a graph learning method on contact maps derived from structures. **GNN-PPI** is a graph neural network-based method that integrates the correlation between proteins and protein sequence features to predict PPIs. **HIGH-PPI** is a hierarchical graph learning model with a dual-view structure, where a node in the PPI network (top outside-of-protein view) is a protein graph (bottom inside-of-protein view).

Supplementary Table 1: Statistics of Protein-Protein, Drug-Protein, and Drug-Drug Interaction Prediction Benchmarks.

Dataset	biomolecules	Splits	Training	Validation	Test
SHS27K	protein: 1553	Random	5328	-	1332
		BFS	4574	-	2086
		DFS	4641	-	2019
BioSNAP	drug: 4510 & target: 2181	Random	9670/9568	1396/1352	2770/2727
		Unseen Protein	9876/9499	1382/1386	2578/2762
		Unseen Drugs	9535/9616	1383/1353	2918/2675
DeepDDI	drug: 1705	Random	153827	19228	19229

Hyperparameters and Supplementary Results

During the EM iterations, we trained the model of atomic structure scale for 30 epochs using a batch size of 8. The initial learning rate was set to 0.001,

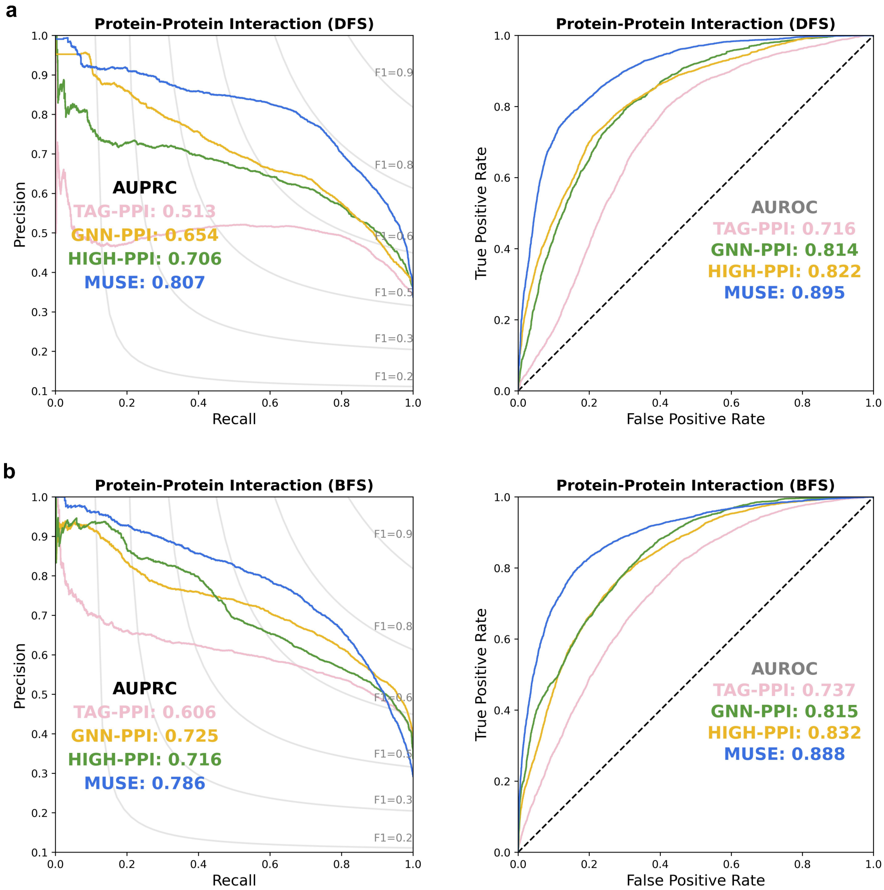

Supplementary Fig. 1: (a) Precision-recall and Receiver Operator characteristic curves of PPI prediction on SHS27k of DFS splits. (b) Precision-recall and Receiver Operator characteristic curves of PPI prediction on SHS27k of BFS splits.

and weight decay was set to 0.0005. The molecular network model was trained for 200 epochs using a batch size of 32 and a learning rate of 0.0001. The iteration k of the EM framework is set to 8 and the pseudo weight is set to 0.2. We use the Adam optimizer during training. Experiments were conducted 5 times, and the mean and standard deviation of AUROC and AUPRC are reported in Table 2. The precision-recall and receiver operator characteristic curves are shown in Figure 1.

Supplementary Table 2: Generalization results on Protein-Protein Interaction and Drug-Protein Interaction predictions.

Method	Random Splits		DFS Splits		BFS Splits	
	AUROC	Best-F1	AUROC	Best-F1	AUROC	Best-F1
DrugVQA	0.852 ± 0.006	0.682 ± 0.004	0.652 ± 0.006	0.533 ± 0.008	0.654 ± 0.005	0.528 ± 0.007
TAG-PPI	0.929 ± 0.007	0.804 ± 0.005	0.716 ± 0.005	0.546 ± 0.007	0.787 ± 0.010	0.530 ± 0.009
GNN-PPI	0.964 ± 0.004	0.854 ± 0.003	0.814 ± 0.004	0.646 ± 0.003	0.815 ± 0.004	0.657 ± 0.005
HIGH-PPI	0.956 ± 0.005	0.883 ± 0.006	0.822 ± 0.004	0.666 ± 0.006	0.832 ± 0.009	0.636 ± 0.008
MUSE	**0.971 ± 0.003**	**0.952 ± 0.004**	**0.888 ± 0.004**	**0.753 ± 0.002**	**0.895 ± 0.001**	**0.725 ± 0.003**
Method	Random Splits		Unseen Protein		Unseen Drugs	
	AUROC	AUPRC	AUROC	AUPRC	AUROC	AUPRC
GNN-CPI	0.866 ± 0.015	0.871 ± 0.014	0.795 ± 0.004	0.748 ± 0.011	0.844 ± 0.015	0.786 ± 0.013
DeepConv	0.883 ± 0.001	0.889 ± 0.005	0.766 ± 0.022	0.723 ± 0.004	0.847 ± 0.009	0.792 ± 0.003
MolTrans	0.895 ± 0.002	0.900 ± 0.014	0.770 ± 0.029	0.739 ± 0.021	0.853 ± 0.011	0.807 ± 0.021
ConPLex	0.897 ± 0.011	0.904 ± 0.014	0.842 ± 0.006	0.754 ± 0.005	0.874 ± 0.002	0.824 ± 0.003
MUSE	**0.915 ± 0.005**	**0.920 ± 0.08**	**0.888 ± 0.003**	**0.791 ± 0.003**	**0.895 ± 0.005**	**0.846 ± 0.006**

1.1.2 Drug-Protein Interactions

Datasets and Baselines

To standardize the drug-protein interactions (DPIs) predictions, we use the DeepDDI benchmark constructed by ConPLex [6], as shown in Table 1. Herein, we consider Unseen drugs and Unseen protein partition schemes, which are variants of the BioSNAP [7] dataset where drugs/proteins in the test set do not appear in any interactions in the training set.

Herein, we include three atomic structure scale models (DeepConv [8], GNN-CPI [9], ConPLex [6]) and a multi-scale model (MolTrans [10]) as our baseline. **DeepConv** employs a convolutional neural network (CNN) on protein sequences and a fully connected layer on molecular fingerprints to predict DPIs. **GNN-CPI** developed a DPI prediction method by combining a graph neural network (GNN) for compounds and a convolutional neural network (CNN) for proteins. **MolTrans** is a Molecular Interaction Transformer on sub-structural patterns and interaction modeling.

Hyperparameters and Supplementary Results

During the EM iterations, we trained the model of atomic structure scale for 50 epochs using a batch size of 8. The initial learning rate was set to 0.0001, and weight decay was set to 0.0005. The molecular network model was trained for 100 epochs using a batch size of 32 and a learning rate of 0.0001. The iteration k of the EM framework is set to 5 and the pseudo weight is set to 0.05. We use the Adam optimizer during training. Experiments were conducted 5 times, and the mean and standard deviation of AUROC and AUPRC are reported in Table 2.

1.1.3 Drug-Drug Interactions

Datasets and Baselines

For the drug-protein interactions (DPIs) predictions, we evaluate the proposed method on the DeepDDI benchmark dataset with different scales for verifying the scalability and robustness of our model. DeepDDI [11] contains 192284 pair-wise DDI and their polypharmacy side-effect information extracted from DrugBank.

Herein, we include two atomic structure scale models (SSI-DDI [12], CGIB [13]) and two multi-scale models (SEAL-CI [14], MIRACLE [10]) as our baseline. **SSI-DDI** is a deep learning framework, which operates directly on the molecular graph representations of drugs for predicting DDIs. **SEAL-CI** firstly applied a hierarchical graph representation learning framework in semi-supervised graph classification tasks, and we use the model to learn drug representations for DDI predictions as a baseline. **MIRACLE** captures the inter-view molecule structure and intra-view interactions between molecules through a contrastive learning scheme. **CGIB** predicts the interaction behavior between a pair of molecular graphs by detecting core subgraphs.

Hyperparameters and Supplementary Results

During the EM iterations, we trained the model of the atomic structure scale for 30 epochs using a batch size of 2048. The initial learning rate was set to 0.001, and weight decay was set to 0.0005. The molecular network model was trained for 100 epochs using a batch size of 1024 and a learning rate of 0.0001. The iteration k of the EM framework is set to 5 and the pseudo weight is set to 0.05. We use the Adam optimizer during training. Experiments were conducted 5 times, and the mean and standard deviation of AUROC and AUPRC are reported in Table 3.

Supplementary Table 3: Results on Drug-Drug Interaction Predictions

Method	Random Splits	
	AUROC	AUPRC
SEAL-CI	0.864 ± 0.022	0.853 ± 0.008
SSI-DDI	0.871 ± 0.011	0.868 ± 0.009
MIRACLE	0.895 ± 0.009	0.944 ± 0.006
CGIB	0.961 ± 0.013	0.950 ± 0.004
MUSE	$\mathbf{0.993 \pm 0.003}$	$\mathbf{0.998 \pm 0.003}$

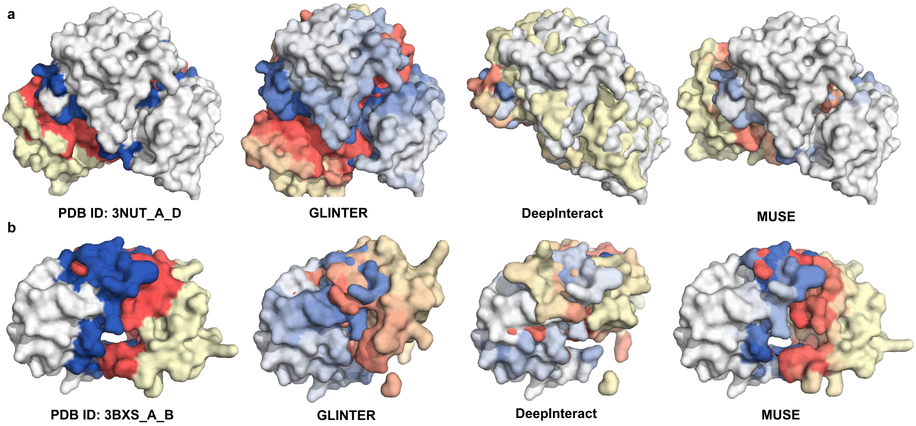

Supplementary Fig. 2: The ground-truth and predictions for PDB entry 3NUT (a) and 3BXS (b). For each method, we show the surface of the predicted and ground truth ligand relative to the ground truth receptor.

1.2 Protein Interface Contact Prediction

1.2.1 Implementation details

For all experiments conducted on protein interface contact prediction, we used two layers of the geometric graph neural network chosen for the atomic structure scale model, which used an edge geometric neighborhood of size $n = 2$ for each edge such that each edge's geometric features are updated by their 4-nearest incoming edges. In addition, we used the Adam optimizer, a learning rate of 0.001, a weight decay rate of 0.001, a dropout (i.e., forget) rate of 0.2, and a batch size of 1 on the NVIDIA A800 GPUs. The molecular network scale model employed a geometric graph neural network model for feature extraction and a ResNet architecture as the interaction module in interface prediction. The interaction module has 14 layers with 64 kernels of size 3×3. Ultimately, the output of the interaction module is a probability-valued A $\times$ B matrix that can be viewed as an inter-chain residue binding heatmap.

1.2.2 Supplementary Results

As shown in Supplementary Fig. 2, MUSE accurately predicts both interfaces while the competing methods fail.

1.3 Protein Binding sites Prediction

1.3.1 Implementation details

For the protein binding sites prediction, we utilized and loaded the geometric graph network network PeSTo [15] as our atomic structure scale model, which was pre-trained on NVIDIA A100 GPUs. The input features are embedded

Supplementary Table 4: Detailed comparison of MUSE with competing methods. A detailed comparison is also performed for all four different testing sets with different cutoff criteria (e.g. sequence identity of at least 70% (1), Homology (2), Topology (3), and None (4)) as defined by the ScanNet[16].

Method	Test (All)		Test (70%)		Test (Homology)		Test (Topology)		Test (None)	
	AUPRC	AUROC	AUPRC	AUROC	AUPRC	AUROC	AUPRC	AUROC	AUPRC	AUROC
ScanNet	0.720	0.897	0.782	0.893	0.691	0.887	0.796	0.921	0.512	<u>0.850</u>
PeSTo	<u>0.797</u>	<u>0.929</u>	<u>0.794</u>	<u>0.912</u>	<u>0.753</u>	<u>0.906</u>	<u>0.900</u>	**0.971**	<u>0.587</u>	0.842
MUSE	**0.811**	**0.938**	**0.823**	**0.930**	**0.804**	**0.935**	**0.891**	<u>0.970</u>	**0.681**	**0.891**

to an input state size of $S = 32$ with a 3-layer neural network with a hidden layer size of 32. Each geometric transformer is composed of 5 neural networks of 3 layers to perform the multi-head self-attention. As for the molecular scale model, we applied a geometric graph neural network for feature extraction of protein structures and a fully connected layer for binding site predictions. Herein, we concatenated the pairwise features to predict the residues whether are binding. To avoid information leakage, the evaluation benchmarking was performed using structures taken from the testing dataset exclusively, removing those structures that appear in the training set of PeSTo. The validation and test examples are also grouped into four subgroups based on their degrees of homology, following the strategy of ScanNet [16].

1.3.2 Supplementary Results

We reported the median receiver operating characteristics (ROC) area under the curve (AUC), the median precision-recall (PR) area under the curve (AUC), and the detailed results are reported in Table 4. And Fig. 3 has demonstrated that MUSE could accurately identify the residues belonging to the binding surface.

2 Ablation study

2.1 Effect of Multi-scale training paradigms.

Figure 4 (a) shows the effect of the different training paradigms on multi-scale learning. One paradigm involves using atomic structure scale information directly as input for the molecular network scale model (denoted as **static**). Another approach is to concatenate the multi-scale representations for label predictions (denoted as **fusion**). Additionally, several **jointly learning** paradigms for fusing the atomic structure scale and molecular network scale have been proposed, including **contrastive learning** and **multi-view** strategy, to effectively integrate multi-scale information for predictions. In this section, we compare our proposed paradigm (MUSE) against the others. We could see that **static** and **fusion** training paradigms exhibit poor efficiency and effectiveness due to their limited ability to integrate multi-scale information. On the other hand, the joint training paradigm (including **contrastive**

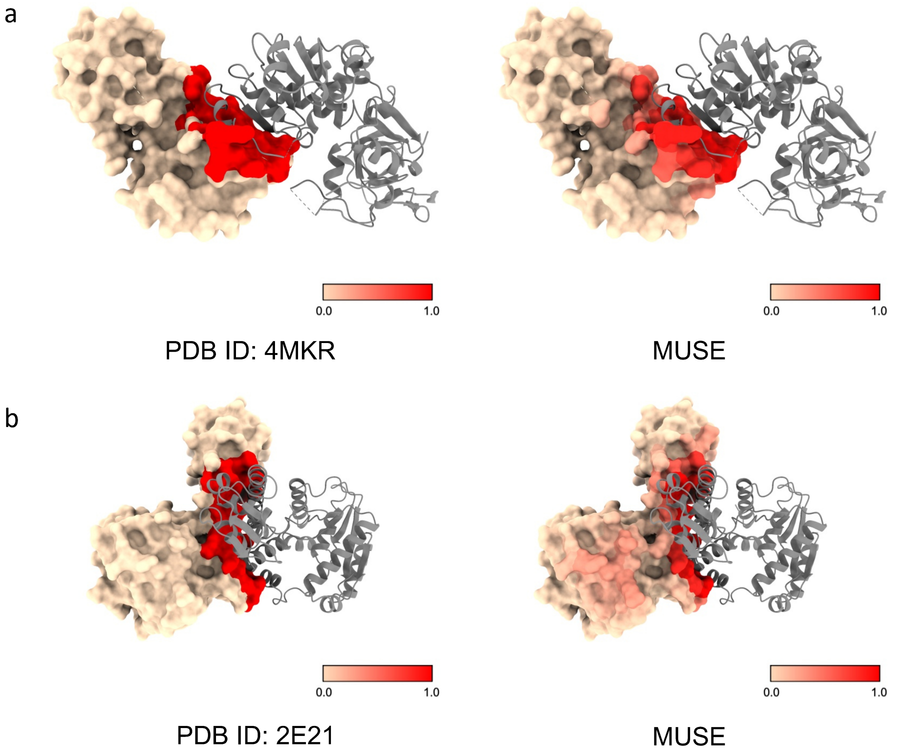

Supplementary Fig. 3: The ground-truth and predictions for PDB entry 4MKR (a) and 2E21 (b).

learning and **multi-view**) offers only marginal improvements due to the imbalanced nature and inherent greediness of multi-scale learning. Finally, our proposed paradigm achieves superior performance by effectively promoting the collaboration of multi-scale information.

2.2 Effect of model architectures.

In Figure 4 (b), we investigate the impact of different GNN model architecture choices on the protein-protein interaction prediction task. To ensure consistent comparison with competing methods (HIGH-PPI [2]), we first employ a graph convolutional network (GCN) to learn structural representations and a graph isomorphism network (GIN) to learn molecular network representations. And we implement GNN variants (GIN[17], GeoGNN [18, 19]) at the intra-molecule scale, and GNN variants (GIN[17], NCN[20]) at the inter-molecule scale for evaluation. We note that the combination of GeoGNN in the atomic structure scale and NCN in the molecular network scale achieves the best performance.

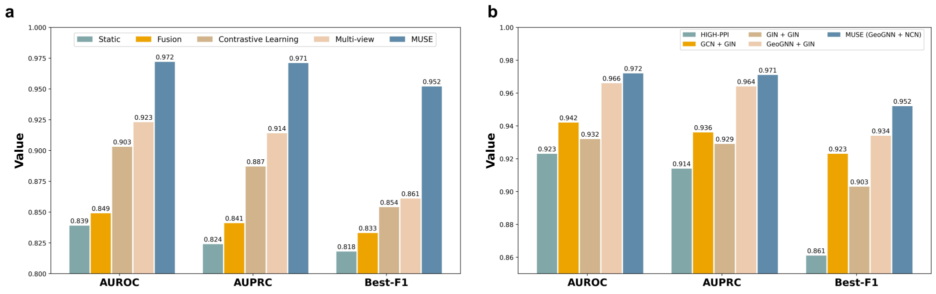

Supplementary Fig. 4: (a) Effect of Multi-scale training paradigms and (b) effect of GNN model architectures.

As shown in Table 5, at the structural scale, the inclusion of ESM2 (MUSE-structure w/ ESM2) could significantly improve PPI predictions over MUSE-structure (w/ GNN). At the network scale, ESM2 led to improved performance but the improvement is smaller. When fusing the structural and the network scale, ESM2 didn't bring improvements in performance but converged faster (converged in 5 iterations).

2.3 Ablation study at different scales

As shown in Table 6, MUSE-structure, which solely focuses on the atomic structure scale, achieved the poorest performance. The MUSE-network outperformed MUSE-structure, demonstrating that the natural information imbalance of different scales exists in the current dataset. MUSE-joint, which integrates two scale models and optimizes them jointly with multiple iterations, showed substantial improvements over the MUSE-structure and MUSE-network. Furthermore, our model MUSE also showed improvements over the ablation study MUSE-joint because of its efficient utilization for multi-scale learning with the proposed EM framework.

Supplementary Table 5: The performance of MUSE on with different protein representation model.

Method	AUROC	Best-F1
MUSE-structure (w/ GNN)	0.904	0.781
MUSE-structure (w/ ESM2)	0.927	0.831
MUSE-network (w/ GNN embeddings)	0.939	0.921
MUSE-network (w/ ESM2 embeddings)	0.943	0.930
MUSE (w/ ESM2)	0.974	0.950 (in 8-iter)
MUSE (w/ GNN)	0.971	0.952 (in 5-iter)

Supplementary Table 6: The performance of MUSE on with different scales.

Method	AUROC	Best-F1
MUSE-structure	0.904	0.781
MUSE-network	0.932	0.874
MUSE	0.971	0.950

2.4 Evaluation on the predicted structures

As the experimental structures are not always available in real-world scenarios, we also investigated the impact on performance when using predicted structures as input for testing. As expected, the performance of HIGH-PPI and MUSE decreases, because they were trained with high-quality native structures. For example, the Best-F1 of HIGH-PPI for predicting PPI decreases from 0.884 to 0.806, compared to the Best-F1 of 0.940 by MUSE. Therefore, in practical situations where experimental structures are unavailable, our proposed method still significantly outperforms the baseline methods.

Supplementary Table 7: The performance comparison of HIGH-PPI and MUSE on the predicted protein structures.

Method	AUROC	Best-F1
HIGH-PPI (AlphaFold)	0.903	0.806
HIGH-PPI (PDB)	0.956	0.884
MUSE (AlphaFold)	0.964	0.940
MUSE (PDB)	**0.971**	**0.952**

References

[1] Qu, M., Bengio, Y., Tang, J.: Gmnn: Graph markov neural networks. In: International Conference on Machine Learning, pp. 5241–5250 (2019). PMLR

[2] Gao, Z., Jiang, C., Zhang, J., Jiang, X., Li, L., Zhao, P., Yang, H., Huang, Y., Li, J.: Hierarchical graph learning for protein–protein interaction. Nature Communications **14**(1), 1093 (2023)

[3] Lv, G., Hu, Z., Bi, Y., Zhang, S.: Learning unknown from correlations: graph neural network for inter-novel-protein interaction prediction. arXiv preprint arXiv:2105.06709 (2021)

[4] Zheng, S., Li, Y., Chen, S., Xu, J., Yang, Y.: Predicting drug–protein interaction using quasi-visual question answering system. Nature Machine Intelligence **2**(2), 134–140 (2020)

[5] Song, B., Luo, X., Luo, X., Liu, Y., Niu, Z., Zeng, X.: Learning spatial structures of proteins improves protein–protein interaction prediction. Briefings in bioinformatics **23**(2), 558 (2022)

[6] Singh, R., Sledzieski, S., Bryson, B., Cowen, L., Berger, B.: Contrastive learning in protein language space predicts interactions between drugs and protein targets. Proceedings of the National Academy of Sciences **120**(24), 2220778120 (2023)

[7] Zitnik, M., Sosic, R., Leskovec, J.: Biosnap datasets: Stanford biomedical network dataset collection. Note: http://snap. stanford. edu/biodata Cited by **5**(1) (2018)

[8] Lee, I., Keum, J., Nam, H.: Deepconv-dti: Prediction of drug-target interactions via deep learning with convolution on protein sequences. PLoS computational biology **15**(6), 1007129 (2019)

[9] Tsubaki, M., Tomii, K., Sese, J.: Compound–protein interaction prediction with end-to-end learning of neural networks for graphs and sequences. Bioinformatics **35**(2), 309–318 (2019)

[10] Huang, K., Xiao, C., Glass, L.M., Sun, J.: Moltrans: molecular interaction transformer for drug–target interaction prediction. Bioinformatics **37**(6), 830–836 (2021)

[11] Ryu, J.Y., Kim, H.U., Lee, S.Y.: Deep learning improves prediction of drug–drug and drug–food interactions. Proceedings of the national academy of sciences **115**(18), 4304–4311 (2018)

[12] Nyamabo, A.K., Yu, H., Shi, J.-Y.: Ssi–ddi: substructure–substructure interactions for drug–drug interaction prediction. Briefings in Bioinformatics **22**(6), 133 (2021)

[13] Lee, N., Hyun, D., Na, G.S., Kim, S., Lee, J., Park, C.: Conditional graph information bottleneck for molecular relational learning. arXiv preprint arXiv:2305.01520 (2023)

[14] Li, J., Rong, Y., Cheng, H., Meng, H., Huang, W., Huang, J.: Semi-supervised graph classification: A hierarchical graph perspective. In: The World Wide Web Conference, pp. 972–982 (2019)

[15] Krapp, L.F., Abriata, L.A., Cortés Rodriguez, F., Dal Peraro, M.: Pesto: parameter-free geometric deep learning for accurate prediction of protein binding interfaces. Nature Communications **14**(1), 2175 (2023)

[16] Tubiana, J., Schneidman-Duhovny, D., Wolfson, H.J.: Scannet: an interpretable geometric deep learning model for structure-based protein binding site prediction. Nature Methods **19**(6), 730–739 (2022)

[17] Xu, K., Hu, W., Leskovec, J., Jegelka, S.: How powerful are graph neural networks? arXiv preprint arXiv:1810.00826 (2018)

[18] Jing, B., Eismann, S., Suriana, P., Townshend, R.J.L., Dror, R.: Learning from protein structure with geometric vector perceptrons. In: International Conference on Learning Representations (2021). https://openreview.net/forum?id=1YLJDvSx6J4

[19] Yuan, Q., Tian, C., Yang, Y.: Genome-scale annotation of protein binding sites via language model and geometric deep learning. BioRxiv (2023)

[20] Wang, X., Yang, H., Zhang, M.: Neural common neighbor with completion for link prediction. arXiv preprint arXiv:2302.00890 (2023)
