# Supplementary material for: A variational expectation-maximization framework for balanced multi-scale learning of protein and drug interactions
Source: Nat Commun. 2024 May 25;15:4476. doi: 10.1038/s41467-024-48801-4 (PMC11530528; doi:10.1038/s41467-024-48801-4)
Supplement: Supplementary file 2 — Reporting Summary [file 41467_2024_48801_MOESM2_ESM.pdf]

nature portfolio

Corresponding author(s): Yuedong Yang

Last updated by author(s): Apr 30, 2024

Reporting Summary

Nature Portfolio wishes to improve the reproducibility of the work that we publish. This form provides structure for consistency and transparency in reporting. For further information on Nature Portfolio policies, see our Editorial Policies and the Editorial Policy Checklist.

Statistics

For all statistical analyses, confirm that the following items are present in the figure legend, table legend, main text, or Methods section.

n/a	Confirmed	
☐	☒	The exact sample size (*n*) for each experimental group/condition, given as a discrete number and unit of measurement
☐	☒	A statement on whether measurements were taken from distinct samples or whether the same sample was measured repeatedly
☒	☐	The statistical test(s) used AND whether they are one- or two-sided<br>*Only common tests should be described solely by name; describe more complex techniques in the Methods section.*
☒	☐	A description of all covariates tested
☒	☐	A description of any assumptions or corrections, such as tests of normality and adjustment for multiple comparisons
☐	☒	A full description of the statistical parameters including central tendency (e.g. means) or other basic estimates (e.g. regression coefficient) AND variation (e.g. standard deviation) or associated estimates of uncertainty (e.g. confidence intervals)
☒	☐	For null hypothesis testing, the test statistic (e.g. F, t, r) with confidence intervals, effect sizes, degrees of freedom and P value noted<br>*Give P values as exact values whenever suitable.*
☒	☐	For Bayesian analysis, information on the choice of priors and Markov chain Monte Carlo settings
☒	☐	For hierarchical and complex designs, identification of the appropriate level for tests and full reporting of outcomes
☒	☐	Estimates of effect sizes (e.g. Cohen's d, Pearson's r), indicating how they were calculated

Our web collection on statistics for biologists contains articles on many of the points above.

Software and code

Policy information about availability of computer code

Data collection	We collected the data from publicly available datasets and previous paper, including Protein Data Bank v50, AlphaFold v2.3.1, STRING V11.5, UniProt, BIOSNAP(https://snap.stanford.edu/biodata/), DeepDDI (https://github.com/isjakewong/MIRACLE/tree/main/MIRACLE/datachem), DIPS-PLUS (https://github.com/BioinfoMachineLearning/DIPS-Plus), PeSTo (https://github.com/LBM-EPFL/PeSTo/tree/main/data/datasets) and ScanNet (https://github.com/jertubiana/ScanNet/tree/main/datasets).
Data analysis	Our framework was implemented by Python 3.9.16 and the Pytorch 1.12.1 library with torch-geometric 2.3.1. The operating system version is Ubuntu 22.04.2. More detais can be found in the Methods section, supplementary information and the code repository (https://github.com/biomed-AI/MUSE).

For manuscripts utilizing custom algorithms or software that are central to the research but not yet described in published literature, software must be made available to editors and reviewers. We strongly encourage code deposition in a community repository (e.g. GitHub). See the Nature Portfolio guidelines for submitting code & software for further information.

Data

Policy information about availability of data

All manuscripts must include a data availability statement. This statement should provide the following information, where applicable:

- Accession codes, unique identifiers, or web links for publicly available datasets
- A description of any restrictions on data availability
- For clinical datasets or third party data, please ensure that the statement adheres to our policy

The PPI and protein data used in this study are obtained from previous study (HIGH-PPI), which are available in the Zenodo database under accession code https://doi.org/10.5281/zenodo.7213401. The DPI data are obtained from previous study (ConPLex), which are available in github (https://github.com/samsledje/ConPLex_dev/tree/main/dataset/BIOSNAP). The DDI data are obtained from previous study (MIRACLE), which are available in github (https://github.com/isjakewong/MIRACLE/tree/main/MIRACLE/datachem). The DIPS-Plus are obtained from previous study (DeepInteract), which are available in https://github.com/BioinfoMachineLearning/DIPS-Plus. The protein-protein binding sites dataset are obtained from https://github.com/jertubiana/ScanNet/tree/main/datasets.
Source data are provided with this paper.

Research involving human participants, their data, or biological material

Policy information about studies with human participants or human data. See also policy information about sex, gender (identity/presentation), and sexual orientation and race, ethnicity and racism.

Reporting on sex and gender	This is not relevant to our study.
Reporting on race, ethnicity, or other socially relevant groupings	This is not relevant to our study.
Population characteristics	This is not relevant to our study.
Recruitment	This is not relevant to our study.
Ethics oversight	This is not relevant to our study.

Note that full information on the approval of the study protocol must also be provided in the manuscript.

Field-specific reporting

Please select the one below that is the best fit for your research. If you are not sure, read the appropriate sections before making your selection.

[x] Life sciences [ ] Behavioural & social sciences [ ] Ecological, evolutionary & environmental sciences

For a reference copy of the document with all sections, see nature.com/documents/nr-reporting-summary-flat.pdf

Life sciences study design

All studies must disclose on these points even when the disclosure is negative.

Sample size	All the datasets for training and evaluating our model are available from existing databases. We use 5 datasets called SHS27K PPI, BioSNAP DPI, DeepDDI, DIPS-Plus and ScanNet PPBS. The SHS27K PPI dataset contains SHS27k (sub-dataset from STRING containing 6600 multi-type of PPIs and 1533 human proteins with native protein structures. The BioSNAP dataset consists of 13863 drug-target interactions with 4510 drugs and 2181 targets. For the DeepDDI dataset, we used 192,284 pair-wise drug-drug interactions extracted from DrugBank. The DIPS-Plus dataset contains 15,618 and 3,548 binary complexes for training and validation. For the protein–protein binding sites (PPBS) dataset, we obtained 41,466 distinct PDB files, involved in 240,506 protein–protein interfaces.
Data exclusions	No data was excluded from the analyses.
Replication	The reproducibility of the experimental findings can be found in our github (https://github.com/biomed-AI/MUSE)
Randomization	This is not relevant to our study because we did not make quantitative comparisons between groups of samples.
Blinding	This is not relevant to our study because there was no group allocation nor sample comparison.

Reporting for specific materials, systems and methods

We require information from authors about some types of materials, experimental systems and methods used in many studies. Here, indicate whether each material, system or method listed is relevant to your study. If you are not sure if a list item applies to your research, read the appropriate section before selecting a response.

Materials & experimental systems

n/a	Involved in the study
☒	☐ Antibodies
☒	☐ Eukaryotic cell lines
☒	☐ Palaeontology and archaeology
☒	☐ Animals and other organisms
☒	☐ Clinical data
☒	☐ Dual use research of concern
☒	☐ Plants

Methods

n/a	Involved in the study
☒	☐ ChIP-seq
☒	☐ Flow cytometry
☒	☐ MRI-based neuroimaging

Plants

Seed stocks	*Report on the source of all seed stocks or other plant material used. If applicable, state the seed stock centre and catalogue number. If plant specimens were collected from the field, describe the collection location, date and sampling procedures.*
Novel plant genotypes	*Describe the methods by which all novel plant genotypes were produced. This includes those generated by transgenic approaches, gene editing, chemical/radiation-based mutagenesis and hybridization. For transgenic lines, describe the transformation method, the number of independent lines analyzed and the generation upon which experiments were performed. For gene-edited lines, describe the editor used, the endogenous sequence targeted for editing, the targeting guide RNA sequence (if applicable) and how the editor was applied.*
Authentication	*Describe any authentication procedures for each seed stock used or novel genotype generated. Describe any experiments used to assess the effect of a mutation and, where applicable, how potential secondary effects (e.g. second site T-DNA insertions, mosiacism, off-target gene editing) were examined.*
